# Enhancement the electrochemical conductivity of a modified reduced graphene oxide/calixarene screen-printed electrode using response surface methodology

**DOI:** 10.1371/journal.pone.0234148

**Published:** 2020-06-05

**Authors:** Nor Zawani Mohamed Azman, Putri Nur Syafieqah Zainal, Shahrul Ainliah Alang Ahmad

**Affiliations:** 1 Department of Chemistry, Faculty of Science, Universiti Putra Malaysia, UPM Serdang, Selangor, Malaysia; 2 Institute of Advanced Technology, Universiti Putra Malaysia, UPM Serdang, Selangor, Malaysia; 3 Research centre for water treatment technologies (TeRAS), Universiti Putra Malaysia, UPM Serdang, Selangor, Malaysia; Lawrence Berkeley National Lab, UNITED STATES

## Abstract

In this paper, Response Surface Methodology with central composite design (RSM/CCD) was used to optimize a modified electrode for improved electron transfer rate and electrochemical performance. The modification was done on a screen-printed carbon electrode (SPCE) with reduced graphene oxide (ERGO)/calix [4] arene (ERGOC4-SPCE). The properties of the modified electrodes were analyzed via cyclic voltammetry, Raman spectroscopy, and Fourier-Transform Infrared (FT-IR) spectroscopy. Then, different variables were optimized, namely, the concentration of graphene oxide, GO (A), the number of scan cycles of graphene oxide (B), and the deposition time (C). The effect of the optimized variables on the reduction-oxidation peak current response of the potassium ferricyanide redox system was analyzed. By using statistical analysis, it shows a significant effect of the concentration of GO, the deposition time, and the number of scans cycles on the peak current response. The coefficient of determination (R^2^) value of 0.9987 produced indicated a good fit of the model with experimental finding.

## 1 Introduction

Graphene-based materials exhibit remarkable chemical and physical properties such as great flexibility, high chemical stability, and superior electric and thermal conductivity [1–3] with promising potential in many applications [4]. Reduced graphene oxide (rGO) is a graphene-based material that has been widely used and explored in various fields. This material can be fabricated through various routes such as chemical [4], thermal [5] or electrochemical [6]. Over the past decade, numerous papers have reported on the extensive use of reduced graphene oxide (rGO) in the field of electrochemistry due to its remarkable conductivity, large surface area, and excellent electrochemical performance [7, 8].

The incorporation of reduced graphene oxide (rGO) with macrocyclic compounds such as calixarene has been found to affect the performance of electrochemical biosensors due to the synergetic effects between rGO and macrocyclic compounds. It has been reported that calix [4] arene can endow RGO with remarkable selectivity and sensitivity towards various analytes [7, 9]. Recently, Zhang et al. reported using electrochemical sensors coupled with RGO-calix [4–8] arene on a glassy carbon electrode to determine tryptophan, ascorbic acid, and dopamine content^7^. The optimum conditions of the modified electrode enhanced the sensitivity of the sensor [10].

Traditionally, analytical chemistry optimization can enhance an experimental response via the monitoring of one factor’s influence at a time. This optimization technique is termed one-variable-at-a-time, as the other variables are kept constant while only one parameter is changed [11]. Jian et al. successfully optimized the accumulation potential and accumulation time of ERGO on the surface of SPCE by testing a standard solution containing 50 ppb Pb^2+^. The study reported an optimal accumulation potential and time of −12 V and 420 s, respectively. However, a major disadvantage of this optimization technique is that the interactive effects between the variables studied are not included. As a consequence, this technique does not depict the complete effects of the parameter on the response. Furthermore, the method cannot describe the interaction between parameters, which could help optimize experimental parameters and provide statistical models [12]. Besides, this technique requires more experiments to conduct research, in turn, leading to increased time and expenses, as well as increased consumption of reagents and materials. Hence, a multivariate statistical approach such as Response Surface Methodology (RSM) is normally used to better understand the optimization process. Response Surface Methodology (RSM) is one of the most relevant multivariate techniques used in analytical optimization. Response Surface Methodology (RSM) is a collection of statistical and mathematical methods used to optimize the response, under the influence of several independent variables [10, 12, 13]. Not many studies have reported on the statistical optimization of electrochemical sensors. Mirmoghtadaie et al. applied statistical factional and factorial design methods to optimize effective parameters to fabricate modified electrodes and immobilize DNA probes [10]. A recent report published the reliability of RSM as an optimization tool to enhance the current signal of clenbuterol based on a poly(3,4-ethylenedioxythiophene) (PEDOT)/multi-walled carbon immunosensor [14]. The optimization of amperometric biosensor performance for detection of Al^3+^ and Bi^3+^ using RSM was explored recently which has demonstrated high sensitivity and better reproducibility, stability and reversibility of developed sensor [15].

The present paper discusses the use of RSM to optimize the fabrication of a modified screen-printed electrode made from calix [4] arene/reduced graphene oxide (ERGOC4-SPCE) to attain the best system performance. The modified electrode was characterized via cyclic voltammetry (CV) and the formation of rGO and calix [4] arene was confirmed using FT-IR and Raman spectroscopy.

## 2 Methodology

### 2.1 Materials and reagents

All chemicals were analytical grade and were used without further purification. Graphene oxide (GO) was purchased from GO Advanced Solution Sdn. Bhd. Meanwhile, 4-tert-butylcalixa [4] rene (C4) was purchased from Aldrich. Potassium ferricyanide (K_3_[Fe(CN)_6_]) and potassium chloride (KCl) were obtained from Bendosen and R&M Marketing (UK), respectively. Deionized water (18.2 MΩ.cm at 25°C, Milli-Q) was used throughout the experiments. Screen-printed electrodes (SPEs) were purchased from DS Dropsens, Spain.

### 2.2 Preparation of GO-SPCE

A GO suspension was previously prepared according to the literature [16] with slight modifications. The graphene oxide solution was first dispersed in a 0.067 M phosphate buffer solution and sonicated for 2 h to achieve a homogeneous suspension. Then, 5 μL of the graphene oxide suspension was dropped onto the surface of the screen-printed carbon electrode (SPCE) and left for 1 h before further use.

### 2.3 Preparation of ERGOC4-SPCE

In this step, 0.2 g/L of 4-tert-butylcalixa [4] rene (C4) was prepared in chloroform. Next, 5 μL of C4 stock solution was dropped onto GO-SPCE and left to dry after which the electrochemical reduction of GO/Calix 4-SPCE was carried out by scanning from -1.4 V to 0 V with a scan rate of 50 mV/s with a varied number of cycles using cyclic voltammetry (ERGO/C4-SPCE). Then, the ERGO/C4-SPCE was carefully washed with distilled water and left to dry in air.

### 2.4 Experimental design and optimization via Response Surface Methodology

Response Surface Methodology based on Central Composite Design was used to evaluate the combined effects of the calix [4] arene deposition time (A), the concentration of GO (B), and the number of cycles of reduced GO (C) on the peak current (the response). The experimental conditions of these factors, which were derived from CCM, are summarized in Table 1. The research variables selected are those that have significant influence to the fabrication of ERGOC4-SPCE and the value range was selected based on preliminary studies (data not shown).

**Table 1:** Experimental range and independent variable level.

			Actual			Coded level		
Variable/factor	Factor	Unit	Low	Middle	High	Low	Middle	High
Deposition time	A	min	40	95	150	-1	0	1
Concentration of GO	B	mg/mL	1.0	1.5	2.0	-1	0	1
Number of scan cycles	C	-	10	15	20	-1	0	1

The response interaction was the studied variables, namely deposition time (A), the concentration of GO (B), and the number of cycles (C), while the response variable was peak current. Central Composite Design allows each independent variable to range from a low level (−1), a central level (0), and a superior level (+1). The RSM/CCD using statistical package software (Design Expert 11.0, Stat Ease Inc., MN, USA) suggested three factors and a total of 20 experimental runs including 8 factorial points, 6 axial points, and 6 central points, obtained using Eq (1):

$$N=2^{n}+2n+n_{c}=2^{3}+2(3)+6=20$$ Eq (1)

Where N is the total number of experiments, n is the number of factors, and n_c_ is the number of replicates in the central point. Response surface modeling, statistical analysis, and optimization were results of the simulations the software. Analysis of Variance (ANOVA) was used to analyze the output data. The experimental data were then fitted to a second-order polynomial regression model, expressed by Eq (2):

$$Y=\beta_{0}+\sum_{i=1}^{k}\beta_{i}x_{i}+\sum_{i=1}^{k}\beta_{ii}{x_{i}}^{2}+\sum_{i=1}^{k-1}\sum_{j>i}^{k}\beta_{ij}x_{i}x_{j}$$ Eq (2)

Where *Y* is the response variable, *x*_*i*_ and *x*_*j*_ are the real or coded variables, and *β*_*0*_, *β*_*i*_, *β*_*ii*_, and *β*_*ij*_ are the regression coefficients where *β*_*0*_ is a constant term, *β*_*i*_ is a linear effect term, *β*_*ii*_ is a quadratic effect term, and *β*_*ij*_ is the interaction effect term. Table 2 shows the design-of-experiment together with the experimental results.

**Table 2:** Central composite design (CCD) for the electrochemical reduction of ERGOC4 using RSM and the experimental peak current response data.

Run	Actual independent variable			Peak current response	
	Deposition time (min)	Concentration of GO (mg/mL)	Number of scan cycles	Actual Value	Predicted Value
1	40	2	10	31.15	30.51
2	150	1	10	41.10	40.68
3	95	1.5	15	71.28	70.79
4	150	2	10	61.09	60.86
5	150	1	20	46.27	46.91
6	95	1.5	23.409	52.41	51.62
9	95	2.3409	15	53.57	53.83
10	95	1.5	6.59104	39.59	40.38
11	95	1.5	15	70.06	70.79
12	40	1	10	42.47	42.45
13	150	2	20	66.85	66.87
14	95	1.5	15	70.24	70.79
15	95	1.5	15	71.62	70.79
16	95	1.5	15	70.67	70.79
17	40	1	20	49.57	49.81
18	95	0.659104	15	47.34	47.08
19	40	2	20	37.23	37.65
20	95	1.5	15	70.89	70.79

### 2.5 Characterizations

The modified electrodes were characterized using an AUTOLAB Type III Model instrument (Eco Chemie B. V., Netherlands). The Ag/AgCl (3.0 M KCl) reference and the platinum electrode (counter) were required to run the three-electrode system analysis of the modified working electrode. Cyclic voltammograms were analyzed using NOVA 1.11 software. The optimized ERGOC4-SPCE was characterized via cyclic voltammetry (CV) in 5 mM K_3_[Fe(CN)_6_]/0.1 M KCl solution in the potential range of −0.4 to 0.8 V at a scan rate of 50 mV s^-1^. All the electrochemical measurements were taken using an AUTOLAB potentiostat. The peak current value was determined from the cyclic voltammogram. The ERGOC4 obtained was confirmed using FT-IR spectral analysis (Perkin Elmer Spectrum 100) between 400 cm^-1^ and 4000 cm^-1^. Several samples were prepared including ERGO, ERGOC4, and GO/C4. The Raman spectroscopic analysis of the GO and GO composites was carried out using a LabRam HR Raman spectrometer (514.5 nm, Ar + laser). Then, the GO composites were put into a vacuum desiccator to pre-dry for 24 h at 60°C and then degassed for 4 h at 100°C under vacuum.

## 3 Results and discussion

### 3.1 Model fitting and statistical analysis of the result

Response surface modeling was conducted to establish the optimum condition for the independent variables to maximize the peak current. Table 1 was used to evaluate the main interaction between the three actual independent variables into three levels (low, middle, and high) with coded values of (-1, 0, +1) and starting points of ± 1.682 for ± α in the CCD pattern. The 20 experiments and their corresponding responses are presented in Table 2.

Design Expert software was used to perform analysis of variance (ANOVA) for the peak current response and a fitted equation model was constructed. The ANOVA for the model shown in Table 3 indicates that the F-value and lack-of-fit F-value of the model were 661.20 and 0.18, implying the significant model and insignificant lack of fit relative to pure error, respectively. This large value of lack-of-fit F is due to noise in the experiment [13]. The F-value model indicates a significant model term because the p-value (< 0.0001) was less than 0.05, Significant regression and a non-significant lack of fit implies that this model is well-fitted to the experiment. The similar model was further used to monitor the interaction of each variable.

**Table 3:** The ANOVA of the peak current of the response surface reduced cubic model.

Source	Sum of Squares	Degree of Freedom	Mean Squared	F-value	p-value	
Model	3316.14	9	368.46	661.20	< 0.0001	Significant
Residual	4.46	8	0.5573			
Lack of Fit	2.65	3	0.8831	2.44	0.1797	Not significant
Pure Error	1.81	5	0.3617			
Cor. Total	3320.60	17				

The coefficient of determination (R^2^) can be a handy tool to ensure the adequacy of a model (Table 4). The model presented a high coefficient of determination (R^2^-value = 0.9987) per the ANOVA of the quadratic regression model, implying that 99% of the variation in the peak current responses can be explained by the independent variables and only about 1% of the variation cannot be explained by the model. The predicted R^2^ (0.9870) was also in reasonable agreement with the adjusted R^2^ (0.9971). The relationship between the experimental and predicted values of the peak current response was established to have a high R^2^-value, which shows that the experimental values match well with the predicted values [13]. Besides, adequate precision measures showed that the signal-to-noise ratio of greater than 4 is desirable. The result of the adequate precision ratio of the peak current response was 72.3959, indicating that the signal was adequate. At the same time, the coefficient of variation (CV) was very low value (1.35), indicating that the experimental values had very high precision and good reliability.

**Table 4:** The statistical parameters indicating the correlation strength between variables in the model equation obtained from ANOVA.

Type of variable	
Standard deviation	0.7465
Mean	55.19
Coefficient of variation (CV) %	1.35
R^2^	0.9987
Adjusted R^2^	0.9971
Predicted R^2^	0.9870
Adequate Precision	72.3959

The second-order polynomial final equation was presented in terms of coded factors to demonstrate the empirical relationship between the actual independent variables and the response. The second-order polynomial equation is presented by Eq (3) below:

$$Y=70.79+6.86A+2.01B+3.34C+8.03AB-0.2815A-0.0547BC-7.87A^{2}-7.19B^{2}-8.76C^{2}$$ Eq (3)

Where Y is the peak current response and A, B, and C are the coded values of the independent variables, which represent the deposition time (min), the concentration of GO (mg/mL), and the number of scan cycles, respectively. The model terms—A, B, C, AB, A^2^, B^2^, and C^2^—were identified as significant model terms with p-values less than 0.0500 (Table 5). The BC and AC model terms were identified as insignificant since the p-value of these terms was greater than 0.005, implying that there is no mutual interaction between deposition time and the number of scan cycles, and between the concentration of GO and number of scan cycles.

**Table 5:** Coefficients of regression of the model and the significance of the coefficients.

Model term	Coefficient estimate	Degree of freedom	p-value	F-value	Standard error	95% confidence interval	
						Low	High
Intercept	70.79	1	-	661.20	0.3048	70.09	71.49
A	6.86	1	< 0.0001	676.03	0.2639	6.25	7.47
B	2.01	1	< 0.0001	98.630	0.2020	1.54	2.47
C	3.34	1	< 0.0001	273.95	0.2020	2.88	3.81
AB	8.03	1	< 0.0001	925.62	0.2639	7.42	8.64
AC	-0.2815	1	0.3173	1.14	0.2639	-0.8901	0.3271
BC	-0.0547	1	0.8408	0.0430	0.2639	-0.6634	0.5539
A^2^	-7.87	1	< 0.0001	420.50	0.3838	-8.75	-6.98
B^2^	-7.19	1	< 0.0001	1113.57	0.2155	-7.69	-6.69
C^2^	-8.76	1	< 0.0001	1654.28	0.2155	-9.26	-8.27

The BC and AC terms were kept in the model equation although these were insignificant terms, to support the hierarchy. This is because insignificant terms still contribute to the model, as this model design could accurately predict up to 2% RSE according to the model validation. However, insignificant terms can be kept or removed from the model equation depending on their suitability and, commonly, insignificant terms are not removed.

### 3.2 Diagnostics

**Fig 1:**
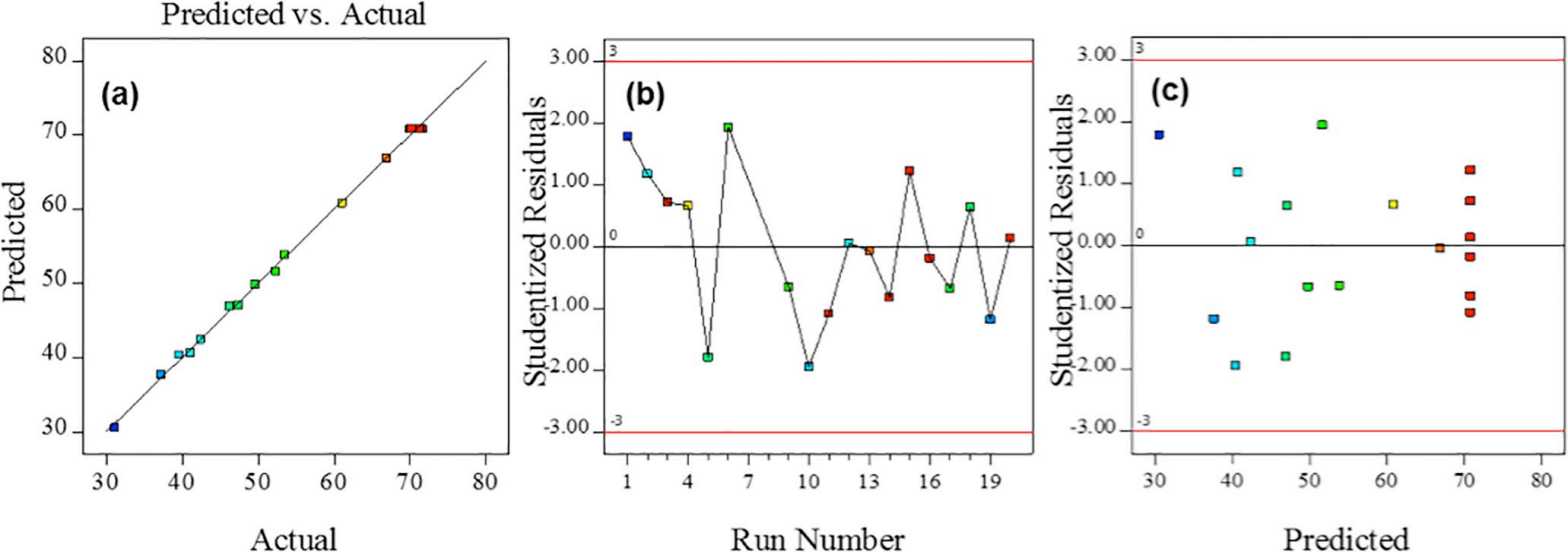
(a) Plot of predicted data versus actual experimental data of the peak current response (b) Plot of studentized residual response versus run number (c) Plot of studentized residual response versus predicted response.

The experimental results of the peak current response obtained versus predicted values were plotted in Fig 1A. The figure shows a linear distribution plot, indicating good correlation between the values and well-fitted model. Based on the adequate correlation, the predictive model can accurately represent the experimental data., The normal probability plot of the studentized residual versus run number was also studied to identify any constant errors that might have occurred (Fig 1B). The figure shows a data was scattered randomly without any trends, which implies no constant error was detected. The plot of standardized residuals versus the predicted response can be used to determine the adequacy of the model. Fig 1C shows the residual response versus the predicted response plot and that the data that was uniformly circulated the mean point of the surface response, implying that this model is an adequate model without any constant error.

### 3.3 Effect of each factor

**Fig 2:**
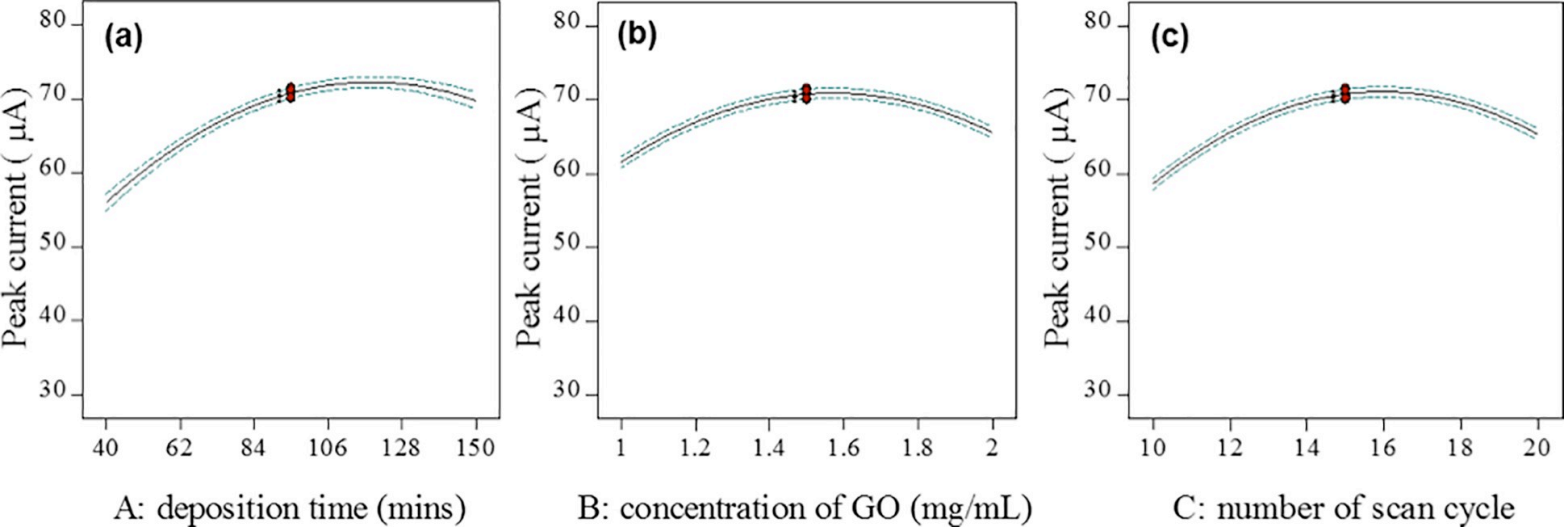
A one-factor plot of the current response as a function of (a) deposition time (concentration of GO = 1.5 mg/mL; number of scan cycles = 15), (b) concentration of GO (deposition time = 95 min; number of scan cycles = 15), and (c) number of scan cycles (concentration of GO = 1.5 mg/mL; deposition time = 95 min).

Fig 2 shows the plots of the effect of each factor (concentration of GO, deposition time, and the number of cycles) on the surface response. Model term A, which represents the deposition time, was significant, per the p-value for model term A that was less than 0.0500. The one-factor plot (Fig 2A) is in agreement with the statement above, where the peak current response increases as the deposition time increases from 40 min to 128 min. However, the peak current decreased with a further increase to 150 min. Meanwhile, an increase in the peak current response was observed with an increased concentration in GO from 1 mg/mL to 1.5 mg/mL (Fig 2B). In comparison to the upper concentration of GO, the peak current was decreased when the concentration of GO further increased to 2 mg/mL. Based on these graphs, the concentration of GO between 1 mg/mL and 2 mg/mL was significant and agrees with the significant p-value calculated using ANOVA. The effect of the plot of the number of reduction cycles (Fig 2C) shows that the peak current response slightly decreased as the number of scan cycles increased from 15 to 20. In comparison to the lower number of scan cycles, more changes in the peak current were observed between 10 and 15 cycles. Therefore, the number of scan cycles is significant when interpreted individually, per its p-value.

### 3.4 Effect of interaction between factors

**Fig 3:**
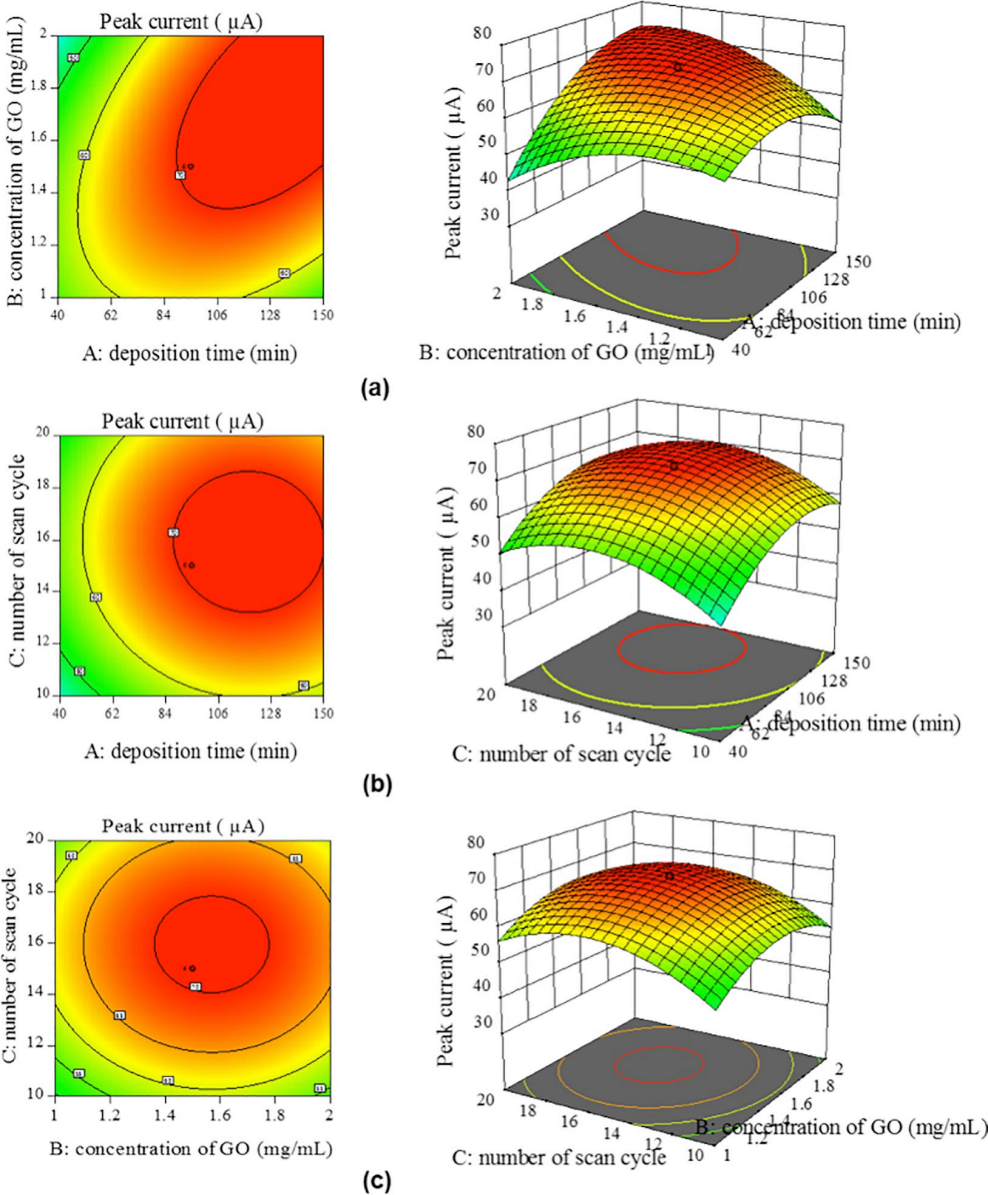
3D surface and 2D contour plots of the peak current as a function of (a) deposition time and concentration of GO, (b) deposition time and the number of scan cycles, and (c) concentration of GO and number of scan cycles.

To obtain a better understanding of the interaction between variables within the range considered, three-dimensional (3D) response surfaces are presented as graphical representations of the regression equation applied [17]. Each contour curve in Fig 3 represents an infinite number of combinations of two significant variables while the other variable is maintained at a respective 0 level. The surface, confined in the smallest ellipse in the contour diagram, indicates the maximum predicted value of the response. Fig 3A reveals the effect of the GO concentration and the deposition time on the peak current response. The peak current response increased when deposition time and GO concentration increased. Fig 3B demonstrates that increased deposition time led to increased peak current up to one point, after which the peak current decreased due to the over-oxidation of GO. Fig 3C shows the effect of GO concentration and the number of cycles on the peak current response. It shows that the highest peak current response occurred when the GO concentration and the number of scan cycles ranged between 1.4 mg/mL and 1.8 mg/mL and 14 and 18 scans, respectively.

### 3.5 Model validation and peak current optimization

A validation test was performed to measure the model validity. In this step, three set experiments were generated from Design-Expert software, that is, to compare the predicted values to the experimental values (Table 6). The maximum peak current response was set as the main goal, while the others were kept in the studied range. The experiments and the response from the optimum combination of the parameters are listed in Table 7. The results show a low residual standard error (RSE) of less than 2%, indicating that the model is valid and can predict the peak current accurately up to 99%.

**Table 6:** Constraints applied for optimization.

Name	Goal	Limit	
		Lower	Upper
Deposition time (min)	Must be in range	40	150
Concentration of GO (mg/mL)	Must be in range	1	2
No. of scan cycles	Must in range	10	20
Peak current (μA)	To maximize	31.149	71.624

**Table 7:** Predicted and observed response values at optimum combinations.

No.	Deposition time (min)	Concentration of GO (mg/mL)	No. of scan cycles	Peak current (μA)		RSE%
				Predicted	Experiment	
1	95	1.618	15	70.8646	71.620	0.805542
2	100	1.618	17	71.5153	70.890	0.804428
3	98	1.618	16	71.6313	70.675	0.805131

### 3.6 Characterizations of ERGOC4-SPCE

#### 3.6.1 Cyclic voltammetry

**Fig 4:**
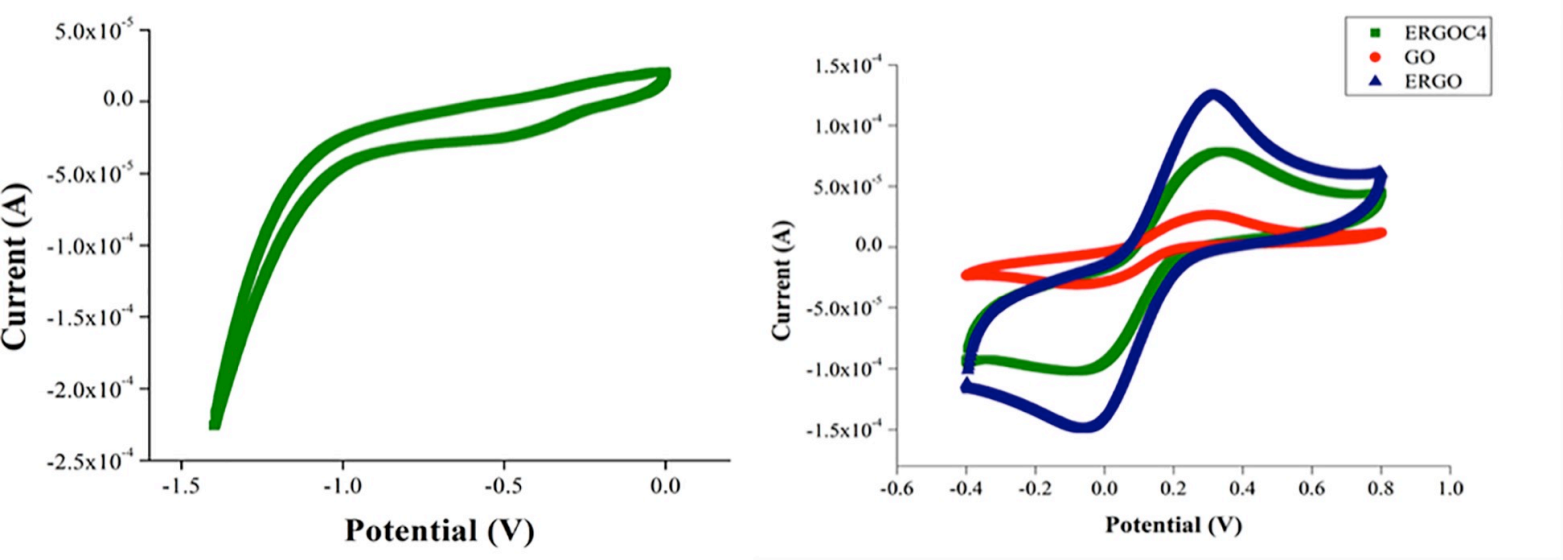
Cyclic voltammograms of (a) electrochemical reduction of GOC4-SPCE in a phosphate buffer solution. (b) GO-SPCE, ERGO-SPCE, GOC4-SPCE, and ERGOC4-SPCE in 0.1 M K_3_[Fe(CN)_6_]/ 0.1 M KCl. Scan rate = 50 mV.

GOC4 was electrochemically reduced via cyclic voltammetry to prepare ERGOC4 on the modified surface of SPCE. Fig 4A shows the cyclic voltammogram of the GOC4-SPCE where a cathodic peak at about -0.5 V appeared in the CV curve, attributed to the irreversible chemical reduction of several oxygen-containing functional groups on the exfoliated GO sheets.

The electrochemical properties of the SPCE before and after the modification were evaluated using a Fe(CN)_6_^3-/4-^ redox probe to verify the interface changes on the surface of SPCE. Fig 4B shows the cyclic voltammograms of the different modified electrodes; GO-SPCE, ERGO-SPCE, and ERGOC4-SPCE. The anodic peak current of ERGO-SPCE (1.07 x 10^−4^ A) increased relative to that of the GO-SPCE (2.62 x 10^−5^ A) due to the large specific surface area of the SPCE after the reduction of GO. The peak-peak potential separation of ERGO-SPCE was 303 mV while GO-SPCE had a 310 mV peak-peak potential separation, indicating a faster electron transfer at the ERGO-SPCE surface. Therefore, it can be concluded that the electrochemical reduction of GO promoted more active sites on the electrode surface, increased the electron transfer, and hence improved the electrochemical properties of the SPCE.

**Fig 5:**
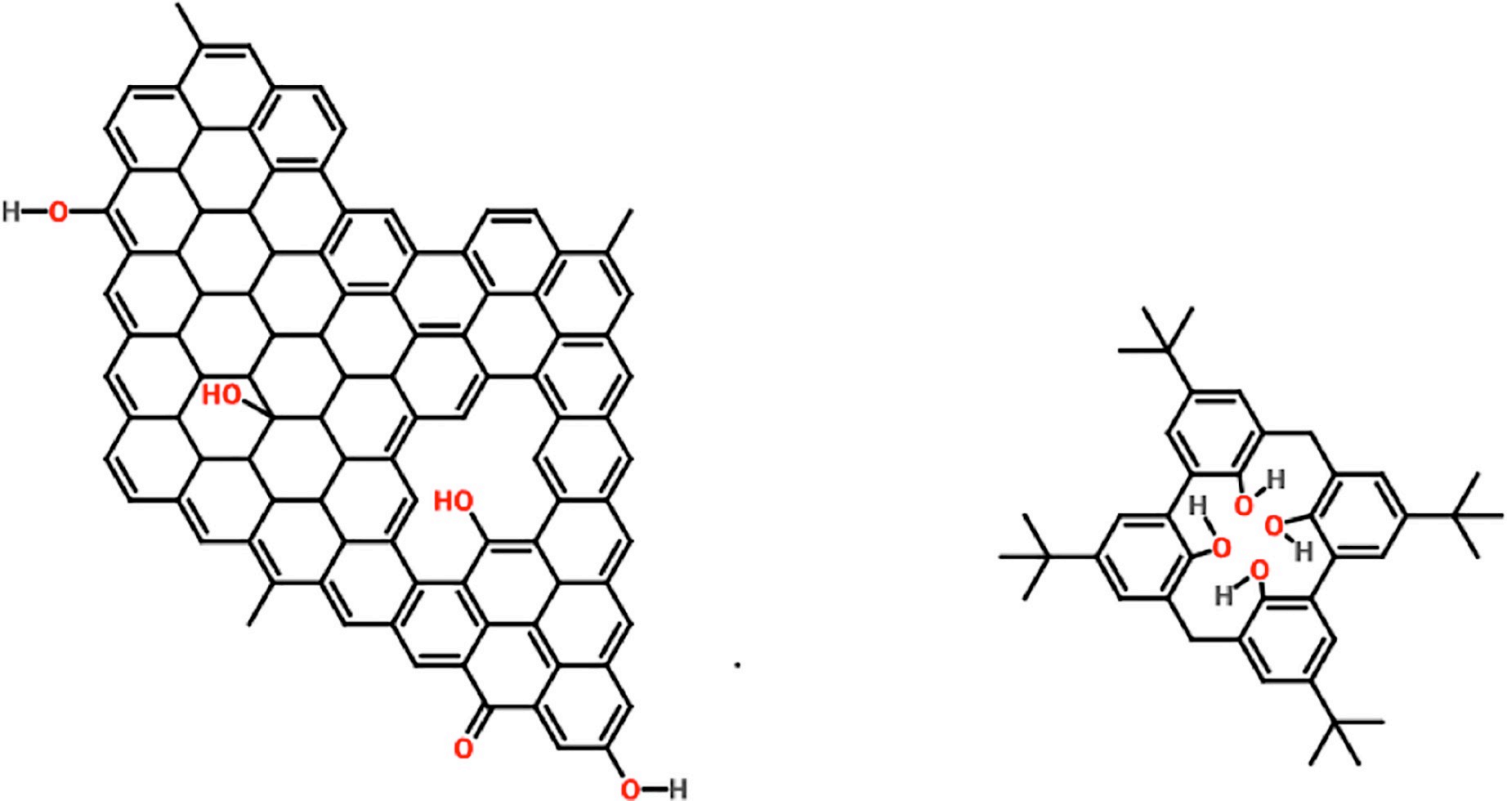
The interaction between ERGO and C4.

However, the redox peak current of ERGOC4-SPCE (7.07 x 10^−5^ A) decreased when calixarene was introduced on the surface of the modified SPCE, indicating that the calixarene, which has a hydrophobic cavity, could not recognize the ions, so the calixarene molecules on the surface of ERGO acted as a layer that blocked the electron transfer between the electrode and Fe(CN)_6_^3-/4-^. This case proves that the calixarenes were successfully immobilized on the SPCE surface. The interaction between some oxygen-containing groups of ERGO and some hydroxyl groups of calixarene was mainly attributed to π-π interactions and hydrogen interactions (Fig 5). Moreover, the hydrophobic cavity of the benzene ring in calixarene could be adsorbed to the ERGO surface via π-π interactions.

#### 3.6.2 Fourier Transform-Infrared Spectroscopy (FTIR)

**Fig 6:**
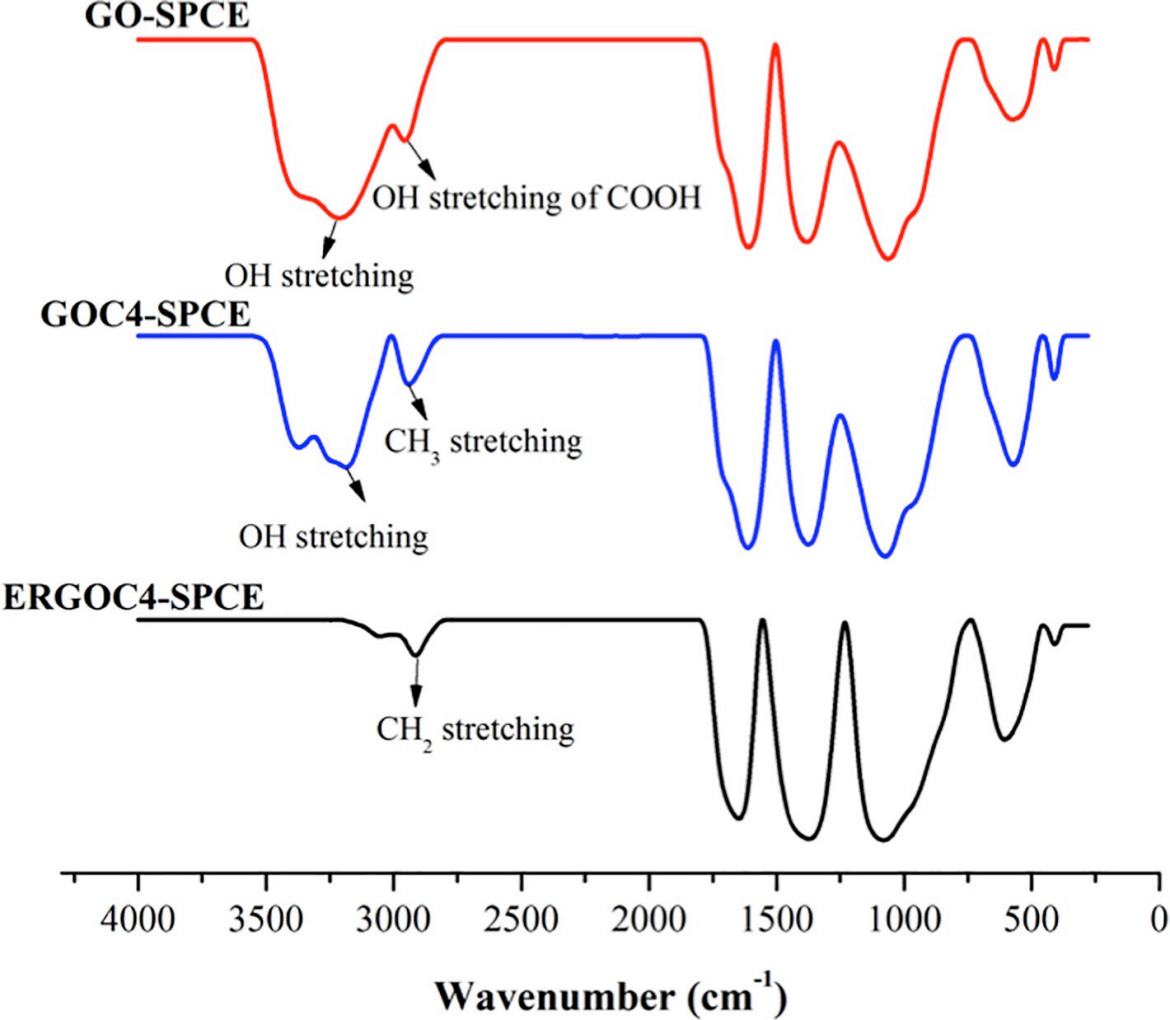
The IR spectra of GO-SPCE, GOC4-SPCE, and ERGOC4-SPCE.

FTIR was conducted to study the functional groups of each modified electrode (GO-SPCE, GOC4-SPCE, and ERGOC4-SPCE), as shown in Fig 6. The spectrum of GO-SPCE depicts peaks at 3213 cm^-1^, 2956 cm^-1^, and 1062 cm^-1^, which are attributed to the carbonyl O-H stretching, the O-H stretching of carboxylic acid, and the C-O stretching vibrations of C-O-C, respectively. Meanwhile, the attachment of C4 onto GO-SPCE shows that the O-H stretching vibration peak shifted from 3213 cm^-1^ to 3186 cm^-1^. Also, a new peak appeared at 2914 cm^-1^ attributed to the CH_2_ vibration in the GOC4 spectra. The shift in the O-H peak might be a result of the typical redshift of noncovalent interaction between GO and C4 through the formation of the hydrogen bond. The IR of the ERGOC4-SPCE preserved all the characteristic bands of GOC4-SPCE except for the O-H stretching peak at 3186 cm^-1^. The peak probably disappeared due to the full attachment of C4 to ERGO-SPCE via hydrogen bonding.

#### 3.6.3 Raman spectroscopy

**Fig 7:**
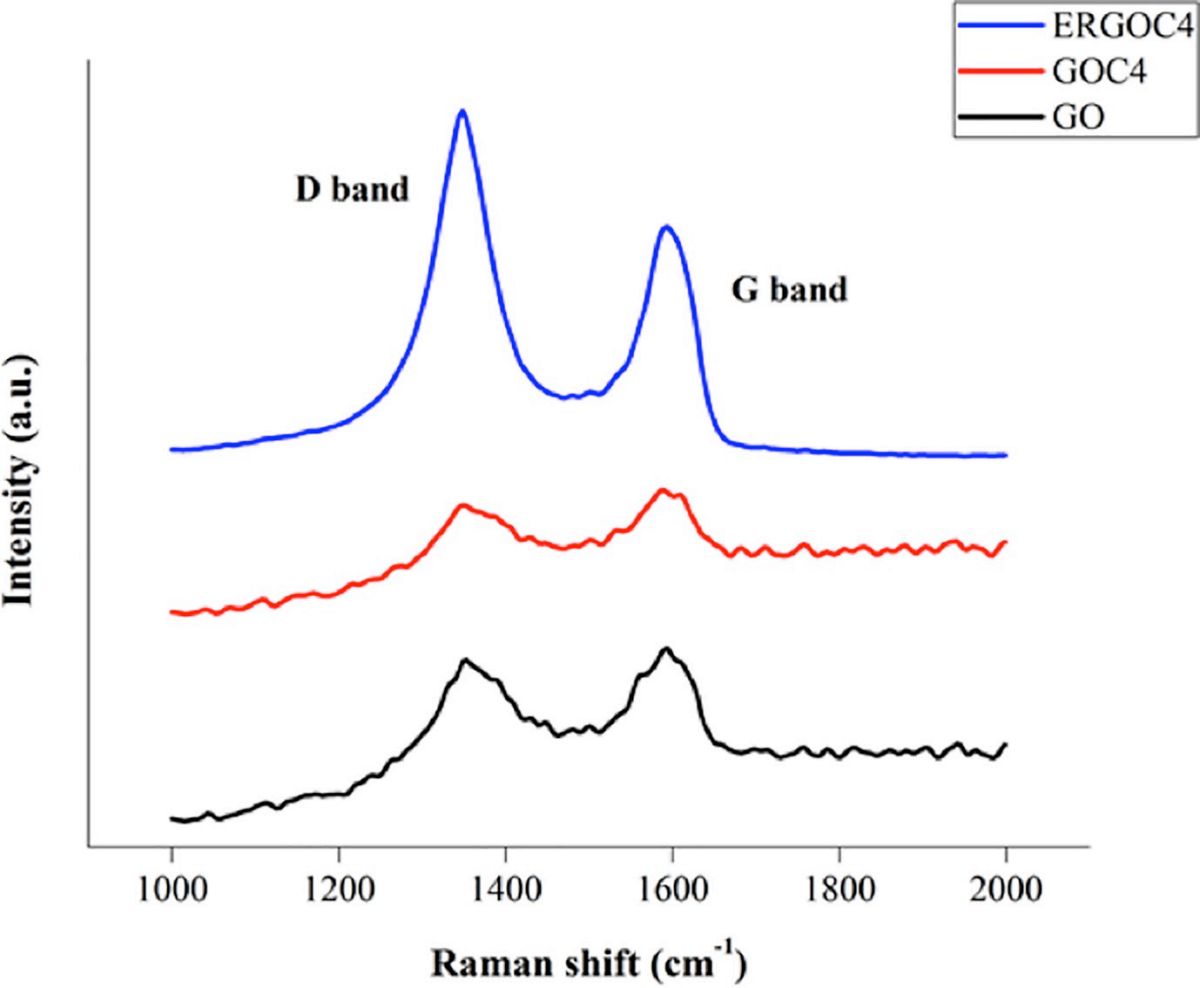
The Raman spectra of GO, GOC4, and ERGOC4 on the surface of SPCE.

Raman spectroscopy was used to characterize the ordered and disordered crystal structure of the carbon materials. In this study, Raman spectra were obtained for GO, GOC4, and ERGOC4 on the surface of SPCE to better understand the change mechanism of the crystal structure, as shown in Fig 7. Two significant bands were observed in the Raman spectrum; D band peaks at 1348.85 cm^-1^ (GOC4 sheet) and 1347.86 cm^-1^ (ERGOC4 sheet), attributed to disorders or defects in the carbon atoms, and G bands at 1588.36 cm^-1^ (GOC4 sheet) and 1596.33 cm^-1^ (ERGOC4 sheet) representing the sp^2^ in-plane vibration of the carbon atoms.

Meanwhile, the intensity ratio of I_D_/I_G_ was determined to define the degree of the disorder and the crystallite size of the graphite material [18]. Table 8 shows the intensity ratio (I_D_/I_G_) of the D band to the G band increased from 0.95 to 1.50 after the electrochemical reduction of GO. Hence, indicating that the reduction had caused a decrease in the size of the in-plane sp^2^ domains and an expansion of the disorder in ERGO. The results also showed shorter and broader D and G bands in ERGOC4-SPCE compared to GO/C4-SPCE, which corresponds to higher electrical conductivity.

**Table 8:** The Raman shift and intensity of GO-SPCE, GOC4-SPCE, and ERGOC4-SPCE.

Materials on the SPCE working electrode	D band		G band		I_D_/I_G_
	Shift (cm^-1^)	Intensity (a.u.)	Shift (cm^-1^)	Intensity (a.u.)	
GO-SPCE	1351.83	0.795	1592.33	0.813	0.98
GOC4-SPCE	1348.85	0.707	1588.36	0.747	0.95
ERGOC4-SPCE	1347.86	0.983	1596.33	0.655	1.50

## 4 Conclusion

In this study, the optimization of peak current response of a reduced graphene oxide (ERGO)/calix [4] arene (C4) composite was successfully performed using response surface methodology (RSM) based on Central Composite Design. The statistical analysis revealed that the concentration of GO, the deposition time, and the number of scan cycles had a significant effect on the peak current response. The R^2^ value of 0.9987 demonstrated a good fit model with the experimental results. Based on the response surface optimization, the optimum condition to obtain the maximum peak current response for the ERGOC4 composite was a GO electrochemical reduction of 1.618 mg/mL, 17 scan cycles, and 100 min deposition time of C4 onto the GO electrode surface. The optimized conditions predicted by the software were compared with the experimental results, and returned a less than 2% error, indicating that the proposed model is reliable and accurately predicted the peak current response. The optimized sensor was characterized using CV and Raman spectroscopy with the results indicating that the GO had been reduced and the calixarene molecules had successfully been introduced onto the surface of GO.

## References

[1] Balandin AA, Ghosh S, Bao W, Calizo I, Teweldebrhan D, Miao F, et al Superior Thermal Conductivity of Single-Layer Graphene. Nano Letters. 2008;8(3):902–7. 10.1021/nl0731872 18284217

[2] Bonaccorso F, Colombo L, Yu G, Stoller M, Tozzini V, Ferrari AC, et al Graphene, related two-dimensional crystals, and hybrid systems for energy conversion and storage. Science. 2015;347(6217):1246501 10.1126/science.1246501 25554791

[3] Xu Z, Gao C. Graphene fiber: a new trend in carbon fibers. Materials Today. 2015;18(9):480–92.

[4] Guex LG, Sacchi B, Peuvot KF, Andersson RL, Pourrahimi AM, Ström V, et al Experimental review: chemical reduction of graphene oxide (GO) to reduced graphene oxide (rGO) by aqueous chemistry. Nanoscale. 2017;9(27):9562–71. 10.1039/c7nr02943h 28664948

[5] Saleem H, Haneef M, Abbasi HY. Synthesis route of reduced graphene oxide via thermal reduction of chemically exfoliated graphene oxide. Materials Chemistry and Physics. 2018;204:1–7.

[6] Tong H, Zhu J, Chen J, Han Y, Yang S, Ding B, et al Electrochemical reduction of graphene oxide and its electrochemical capacitive performance. J Solid State Electrochem. 2013;17(11):2857–63.

[7] Si K, Sun C, Cheng S, Wang Y, Hu W. Cyclodextrin functionalized reduced graphene oxide for electrochemical chiral differentiation of tartaric acid. Analytical Methods. 2018;10(29):3660–5.

[8] Ahmad R, Mahmoudi T, Ahn M-S, Yoo J-Y, Hahn Y-B. Fabrication of sensitive non-enzymatic nitrite sensor using silver-reduced graphene oxide nanocomposite. Journal of Colloid and Interface Science. 2018;516:67–75. 10.1016/j.jcis.2018.01.052 29408145

[9] Zainal PNS, Ahmad SAA, Ngee LH. Surface Modification of Screen-Printed Carbon Electrode (SPCE) with Calixarene-Functionalized Electrochemically Reduced Graphene Oxide (ERGO/C4) in the Electrochemical Detection of Anthracene. J Electrochem Soc. 2019;166(2):B110–B6.

[10] Mirmoghtadaie L, Ensafi AA, Kadivar M, Norouzi P. Highly selective electrochemical biosensor for the determination of folic acid based on DNA modified-pencil graphite electrode using response surface methodology. Materials Science and Engineering: C. 2013;33(3):1753–8. 23827633 10.1016/j.msec.2012.12.090

[11] Lundstedt T, Seifert E., Abramo L., Thelin B., Nystr ¨om A., Pertensen J., et al Experimental design and optimization. Chemometr Intell Lab Syst 42 (1998) 3. 1998;42:3–40.

[12] Alam Z, Muyibi SA, Toramae J. Statistical optimization of adsorption processes for removal of 2,4-dichlorophenol by activated carbon derived from oil palm empty fruit bunches. Journal of Environmental Sciences. 2007;19(6):674–7. 10.1016/s1001-0742(07)60113-2 17969639

[13] Bezerra MA, Santelli RE, Oliveira EP, Villar LS, Escaleira LA. Response surface methodology (RSM) as a tool for optimization in analytical chemistry. Talanta. 2008;76(5):965–77. 10.1016/j.talanta.2008.05.019 18761143

[14] Talib NAA, Salam F, Yusof NA, Alang Ahmad SA, Azid MZ, Mirad R, et al Enhancing a clenbuterol immunosensor based on poly(3,4-ethylenedioxythiophene)/multi-walled carbon nanotube performance using response surface methodology. RSC Advances. 2018;8(28):15522–32. 10.1039/c8ra00109j PMC9088606 35559117

[15] De Benedetto GE, Di Masi S, Pennetta A, Malitesta C. Response Surface Methodology for the Optimisation of Electrochemical Biosensors for Heavy Metals Detection. Biosensors. 2019;9(1):26. 10.3390/bios9010026 PMC6468913 30781820

[16] Jian J-M, Liu Y-Y, Zhang Y-L, Guo X-S, Cai Q. Fast and Sensitive Detection of Pb2+ in Foods Using Disposable Screen-Printed Electrode Modified by Reduced Graphene Oxide. Sensors. 2013;13:13063–75. 10.3390/s131013063 24077322 PMC3859050

[17] Mangili I, Lasagni M, Huang K, Isayev AI. Modeling and optimization of ultrasonic devulcanization using the response surface methodology based on central composite face-centered design. Chemometrics and Intelligent Laboratory Systems. 2015;144:1–10.

[18] Mohan VB, Brown R, Jayaraman K, Bhattacharyya D. Characterisation of reduced graphene oxide: Effects of reduction variables on electrical conductivity. Materials Science and Engineering: B. 2015;193:49–60.

